# The Mutual Regulatory Role of Ferroptosis and Immunotherapy in Anti-tumor Therapy

**DOI:** 10.1007/s10495-024-01988-9

**Published:** 2024-06-09

**Authors:** Zhiguo Mao, Yilong Hu, Yinan Zhao, Xiaolei Zhang, Lin Guo, Xiaoran Wang, Jinying Zhang, Mingsan Miao

**Affiliations:** 1 Department of Pharmacology, Zhengdong New District, Henan University of Chinese Medicine, No. 156 Jinshui East Road, Zhengzhou, 450046 Henan China; 2 Collaborative Innovation Center of Research and Development, Whole Industry Chain of Yu-Yao in Henan Province, Henan, China

**Keywords:** Ferroptosis, Immunotherapy, Tumor, Immune checkpoint, Combined therapy

## Abstract

Ferroptosis is a form of cell death that is triggered by the presence of ferrous ions and is characterized by lipid peroxidation induced by these ions. The mechanism exhibits distinct morphological characteristics compared to apoptosis, autophagy, and necrosis. A notable aspect of ferroptosis is its ability to inhibit uncontrolled tumor replication and immortalization, especially in malignant, drug-resistant, and metastatic tumors. Additionally, immunotherapy, a novel therapeutic approach for tumors, has been found to have a reciprocal regulatory relationship with ferroptosis in the context of anti-tumor therapy. A comprehensive analysis of ferroptosis and immunotherapy in tumor therapy is presented in this paper, highlighting the potential for mutual adjuvant effects. Specifically, we discuss the mechanisms underlying ferroptosis and immunotherapy, emphasizing their ability to improve the tumor immune microenvironment and enhance immunotherapeutic effects. Furthermore, we investigate how immunotherapeutic factors may increase the sensitivity of tumor cells to ferroptosis. We aim to provide a prospective view of the promising value of combined ferroptosis and immunotherapy in anticancer therapy by elucidating the mutual regulatory network between each.

**Graphical Abstract:**

Ferroptosis in the tumor microenvironment involves intricate crosstalk between tumor cells and immune cells. Through MHC recognition, CD8^+^T cells activate the JAK1/STAT1 pathway in tumor cells, impairing the function of System Xc and reducing GSH and GPX4 expression to promote tumor cell ferroptosis. Additionally, activation of the STAT1-IRF1-ACSL4 pathway could also promote ferroptosis. The blockade of the antioxidant pathway in tumor cells induces ferroptosis, and the released DAMPs could promote DCs maturation through the cGAMP-STING-TBK1 pathway, leading to antigen presentation that activates CD8^+^T cells. The release of DAMPs also induces the M1-type polarization of macrophages, which exerts an anti-tumor effect. The anti-tumor effects of CD8^+^T cells could also be enhanced by blocking inhibitory immune checkpoints such as PD-1, PD-L1, CTLA4, and LAG3. Abbreviations: ACSL4, acyl-CoA synthetase long-chain family member 4; BH4, tetrahydrobiopterin; cGAMP, cyclic GMP-AMP; CTLA4, cytotoxic T lymphocyte-associated antigen-4; DCs, dendritic cells; DHFR, dihydrofolate reductase; DHODH, dihydroorotate dehydrogenase; GPX4, glutathione peroxidase 4; GSH, glutathione; HIF-1α, Hypoxia-Inducible Factor-1α;IFN-γ, interferon-γ; IRF1, interferon regulatory factor 1;IRP1, iron regulatory protein 1; JAK 1, janus kinase; LAG3, lymphocyte activation gene 3; MHC, major histocompatibility complex; NRF2, nuclear factor erythroid-2-related factor 2; PD-1, programmed death protein -1; PD-L1, programmed death ligand 1; PUFA, polyunsaturated fatty acid; ROS, reative oxygen species; STAT1, signal transducer and activator of transcription 1; STING, stimulator of interferon genes; TBK1, TANK-binding kinase 1 TLR2, toll-like receptor 2. This diagram was drawn by Figdraw (www.figdraw.com).

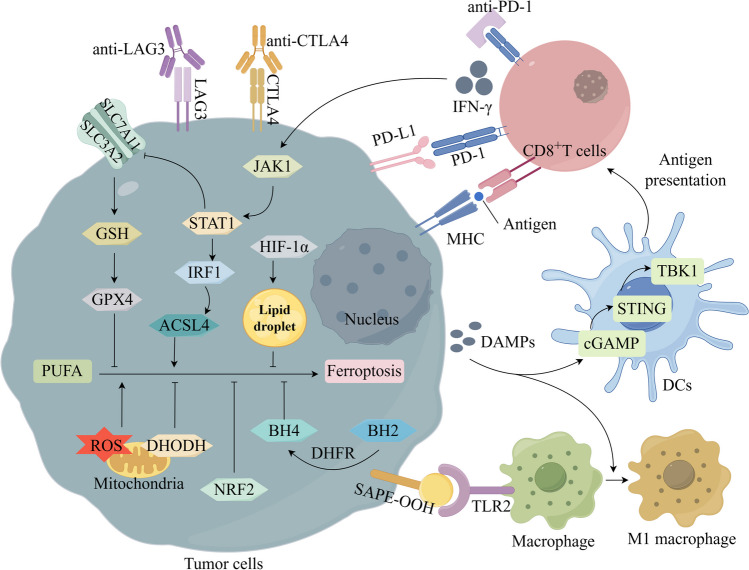

## Introduction

Ferroptosis is an iron-dependent programmed death characterized by lipid peroxidation, proposed by Stockwell and colleagues in 2012 [1]. Ferroptosis exhibits distinct morphological differences from other programmed deaths, such as apoptosis, necrosis, autophagy, etc. These differences include mitochondrial atrophy, a reduced number of mitochondrial cristae, and unique phospholipid oxidation features. The process of ferroptosis is mainly regulated by a combination of intracellular executive systems, such as ferrous ion overload and lipid peroxidation, and the antagonistic effects of defense systems, such as antioxidant systems. The antioxidant system mainly consists of the glutathione peroxidase 4 (GPX4), ferroptosis suppressor protein 1 (FSP1), dihydroorotate dehydrogenase (DHODH), Fas-associated factor 1 (FAF1), and other antioxidant pathways [2]. Upon disruption or insufficient function of the intracellular antioxidant system, ferroptosis is triggered by an accumulation of iron and lipid peroxidation [3]. Ferroptosis is fatal for almost all cells, including tumor cells.

Tumor generation is mainly dependent on the accumulation of mutant proto-oncogenes, which induce metabolic reprogramming of tumor cells, helping them adapt to harsh survival environments such as oxidative stress, accelerating cancer cell proliferation, and also conferring invasive, metastatic, and drug-resistant capabilities [4]. Notably, ferroptosis may be a common endpoint of most aberrant metabolic pathways [5]. Specific genetic mutations, unique metabolic modalities, and high-load oxidative stress all contribute to making tumor cells more susceptible to ferroptosis. Tumor drug resistance can be reversed by targeting ferroptosis susceptibility [6]. Relevant studies have demonstrated that immunotherapy and ferroptosis share a reciprocal regulatory role. Interferon-γ (IFN-γ) derived from CD8^+^T cells can stimulate ferroptosis in cancer cells via polyunsaturated fatty acids (PUFA) [7].

The academic community has been facing a significant challenge in recent years regarding immune tolerance in tumor therapy, which can be co-induced through pathways such as the activation of co-stimulatory factors on T-cell surfaces. To counteract immune tolerance, immunotherapeutic agents, particularly immune checkpoint inhibitors, have been employed [8]. Significantly, ICIs, such as cytotoxic T lymphocyte-associated antigen-4 (CTLA4), programmed death protein-1 (PD-1), and its ligand programmed death ligand 1 (PD-L1), have been found to induce lipid peroxidation and ferroptosis in tumor cells [9]. Studies have also shown that ICIs are hindered by the ferroptosis inhibitor rilastatin 1 [10]. Consequently, exploring the potential strategy of mutual crosstalk between immunotherapy and ferroptosis may hold promise for cancer treatment. A comprehensive overview of the therapeutic network pathways involved in ferroptosis and immunotherapy is presented here. Additionally, we elucidate the interplay between immunotherapy and ferroptosis pathways, highlighting their synergistic effect in targeting and eliminating tumor cells. These findings offer novel insights and potential directions for the development of natural antitumor drugs.

## Tumors and ferroptosis

**Fig. 1:**
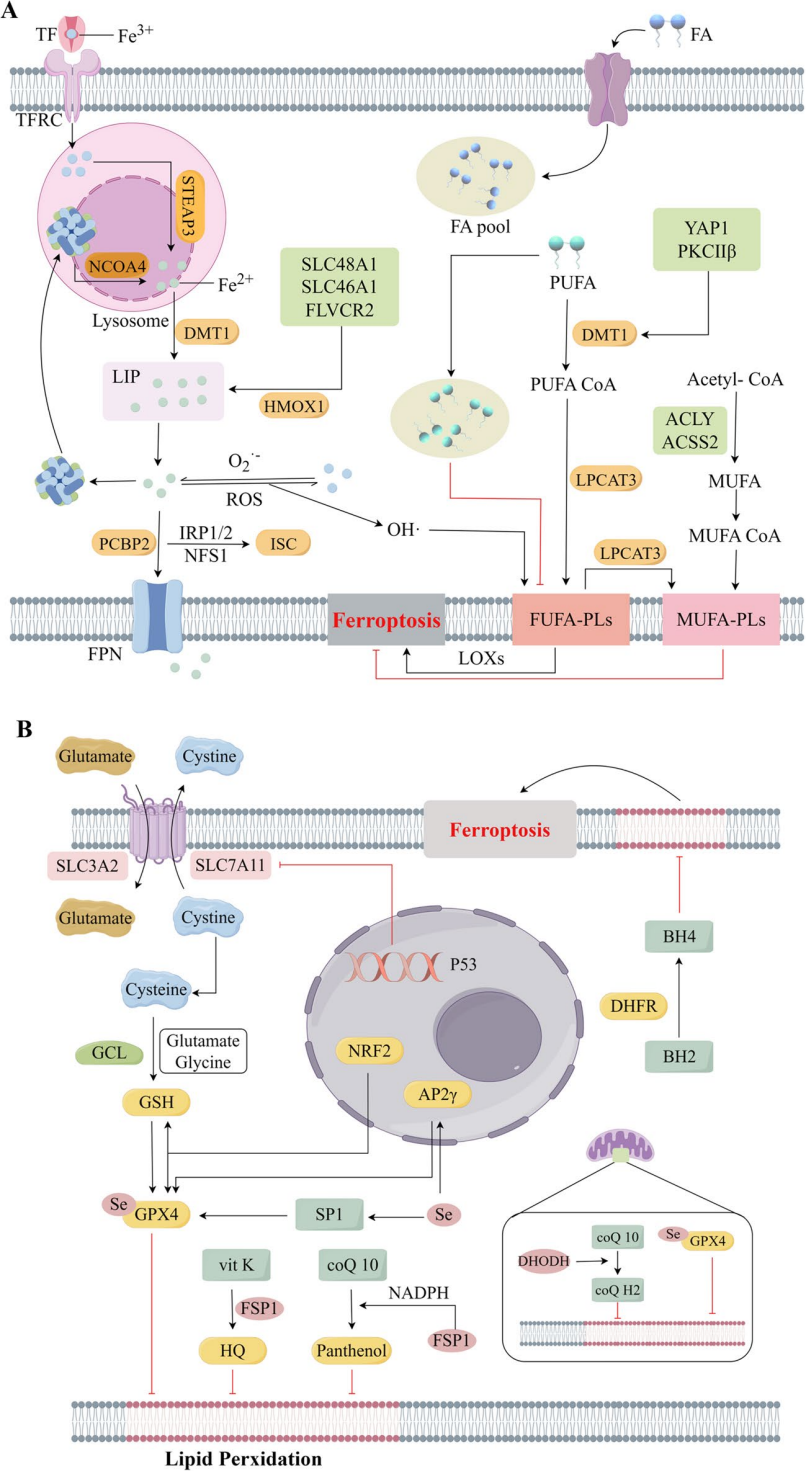
Regulatory mechanisms of ferroptosis. A. Intracellular iron metabolism and lipid metabolism synergistically induce ferroptosis. Fe^3+^ is transported intracellularly via TF and TFRC, and lysosomal digestion mediated by STEAP3 releases Fe^2+^ to replenish the LIP. Fe^2+^ undergoes the Fenton reaction, releasing large amounts of reactive oxygen species (ROS) and free radicals. Fatty acid uptake increases the intracellular pool of fatty acids. PUFA catalyzed by acyl-CoA synthetase long-chain family member 4 (ACSL4) and LPCAT3 form PUFA-phospholipids (PLs), which are susceptible to oxidative stress and promote ferroptosis mediated by lipoxygenases (LOXs). An increase in monounsaturated fatty acid phospholipids (MUFA-PLs) as well as a decrease in polyunsaturated fatty acid phospholipids (PUFA-PLs) inhibits ferroptosis. B. The antioxidant pathway plays a crucial role in inhibiting intracellular ferroptosis. cystine/glutamate transporter (System Xc-) promotes intracellular cysteine uptake and induces the expression of GSH and GPX4, leading to increased antioxidant effects and inhibition of lipid peroxidation in cell membranes. NRF2, AP-2γ, and SP1 all upregulate the expression of GPX4, thereby enhancing the antioxidant effects. Additionally, FSP1 increases intracellular production of HQ and ubiquinol, exerting antioxidant effects. The promotion of BH4 synthesis by DHFR also results in antioxidant effects. Within mitochondria, both DHODH and GPX4 have the capability to inhibit ferroptosis by blocking lipid peroxidation. Abbreviations: ACLY, ATP citrate lyase; ACSS2, Acetyl-CoA synthetase 2; BH4, tetrahydrobiopterin; DHFR, dihydrofolate reductase; DHODH, dihydroorotate dehydrogenase; DMT1, divalent metal-ion transporter-1; FSP1, Ferroptosis suppressor protein 1; GCL, glutamate-cysteine ligase; GPX4, Glutathione Peroxidase 4; GSH, glutathione; HMOX1, heme oxygenase 1; HQ, hydroquinone; IRP1/2, iron regulatory protein 1/2; ISC, iron-sulfur cluster; LIP, labile iron pool; LOXs, lipoxygenases; LPCAT3, lysophosphatidylcholine acyltransferase 3; MUFA, monounsaturated fatty acid; NCOA 4, nuclear receptor coactivator 4; NFS1, nitrogen fixation 1; NRF2, nuclear factor erythroid-2-related factor 2; PCBP2, Poly(rC)-binding Protein 2; PKCβII, protein kinase C βII; PUFA, polyunsaturated fatty acid; SP1, specificity protein 1; STEAP3, six-transmembrane epithelial antigen of prostate 3; TF, transferrin; TFRC, transferrin receptor protein; YAP1, Yes associated protein 1. This diagram was drawn by Figdraw (www.figdraw.com)

**Fig. 2:**
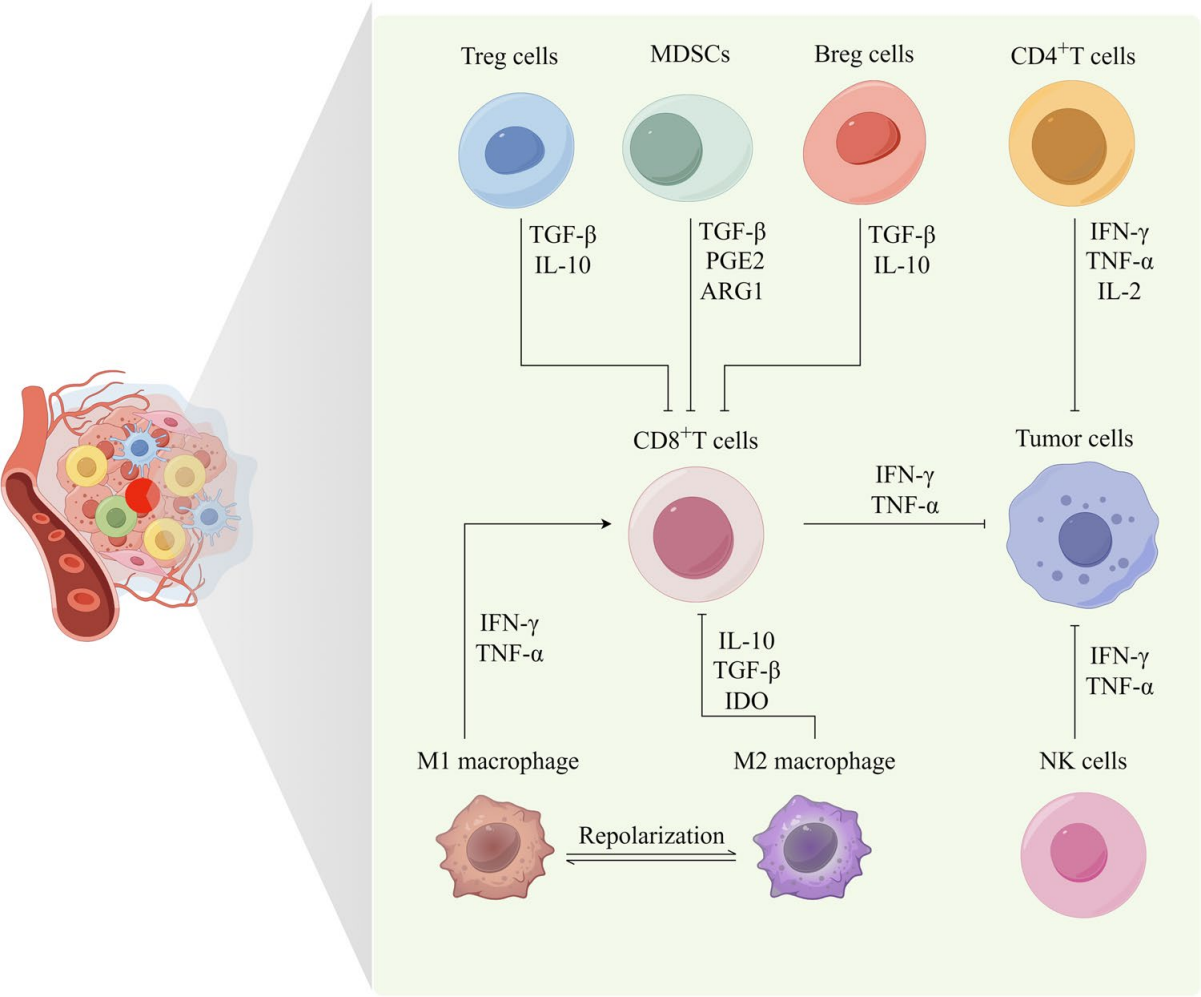
Cell interactions in the tumor microenvironment involve various immune cells. CD8^+^T cells release IFN-γ and tumor necrosis factor α (TNF-α), which leads to the inhibition of tumor cell growth. Similarly, CD4^+^T cells release IFN-γ, interleukin-2 (IL-2), and TNF-α, contributing to the inhibition of tumor cell growth. NK cells release IFN-γ and TNF-α, resulting in the inhibition of tumor cell growth. Tregs release transforming growth factor-β (TGF-β) and IL-10, leading to the inhibition of CD8^+^T cell function. Furthermore, MDSCs release TGF-β, prostaglandin E2 (PGE2), and ARG1, which inhibits CD8^+^T cell function, while Bregs release TGF-β and IL-10, also leading to the inhibition of CD8^+^T cell function. Different polarizations of macrophages in the tumor microenvironment play a significant role in influencing tumors. M1-type macrophages can activate CD8^+^T cells through antigen presentation or secretion of IFN-γ and TNF-α, thereby exerting anti-tumor effects. Additionally, M1-type macrophages can directly kill tumor cells. On the other hand, M2-type macrophages inhibit CD8^+^T cell function by secreting IL-10, TGF-β, and IDO, contributing to the suppression of the immune response. This diagram was drawn by Figdraw (www.figdraw.com)

Among cancer patients with non-small cell lung cancer, breast cancer, and pancreatic cancer, ferroptosis targeted therapy has demonstrated effectiveness. The primary mechanisms driving ferroptosis involve the Fenton reaction brought about by excessive intracellular accumulation of ferrous ions (promoting uptake and inhibiting storage and efflux) and oxidative stress-induced lipid peroxidation of PUFA (Fig. 1). Cisplatin and dihydroartemisinin modulate the transportation and discharge of substantial quantities of Fe^2+^ from lung cancer cells by means of iron-regulatory proteins/iron response elements, thereby initiating ferroptosis [11]. Ficolin 3 enhances ferroptosis in hepatocellular carcinoma by suppressing de novo synthesis of MUFA via down-regulation of the insulin receptor β (IR-β)/sterol regulatory element binding protein-1c (SREBP1c) axis, leading to elevated levels of PUFA-enriched phospholipids and intracellular ROS [12]. In contrast, The intracellular defense against ferroptosis heavily relies on the antioxidant system, this system includes various components such as GPX4, FSP1, DHODH, FAF1, tetrahydrobiopterin (BH4), and nuclear factor erythroid-2-related factor 2 (NRF2) (Fig. 2). Inhibitors such as erastin, temozolomide, and lutazosulfapyridine have been found to decrease system Xc- activity, resulting in reduced cystine uptake and increased generation of ROS, ultimately promoting ferroptosis in tumor cells. With both systems working in tandem to regulate ferroptosis process. Notably, tumor cell ferroptosis is also intricately connected to energy metabolism due to the heightened metabolic requirements for growth. The treatment of ferroptosis of tumor is described in detail in the reference [13].

## Tumor and immunotherapy

**Fig. 3:**
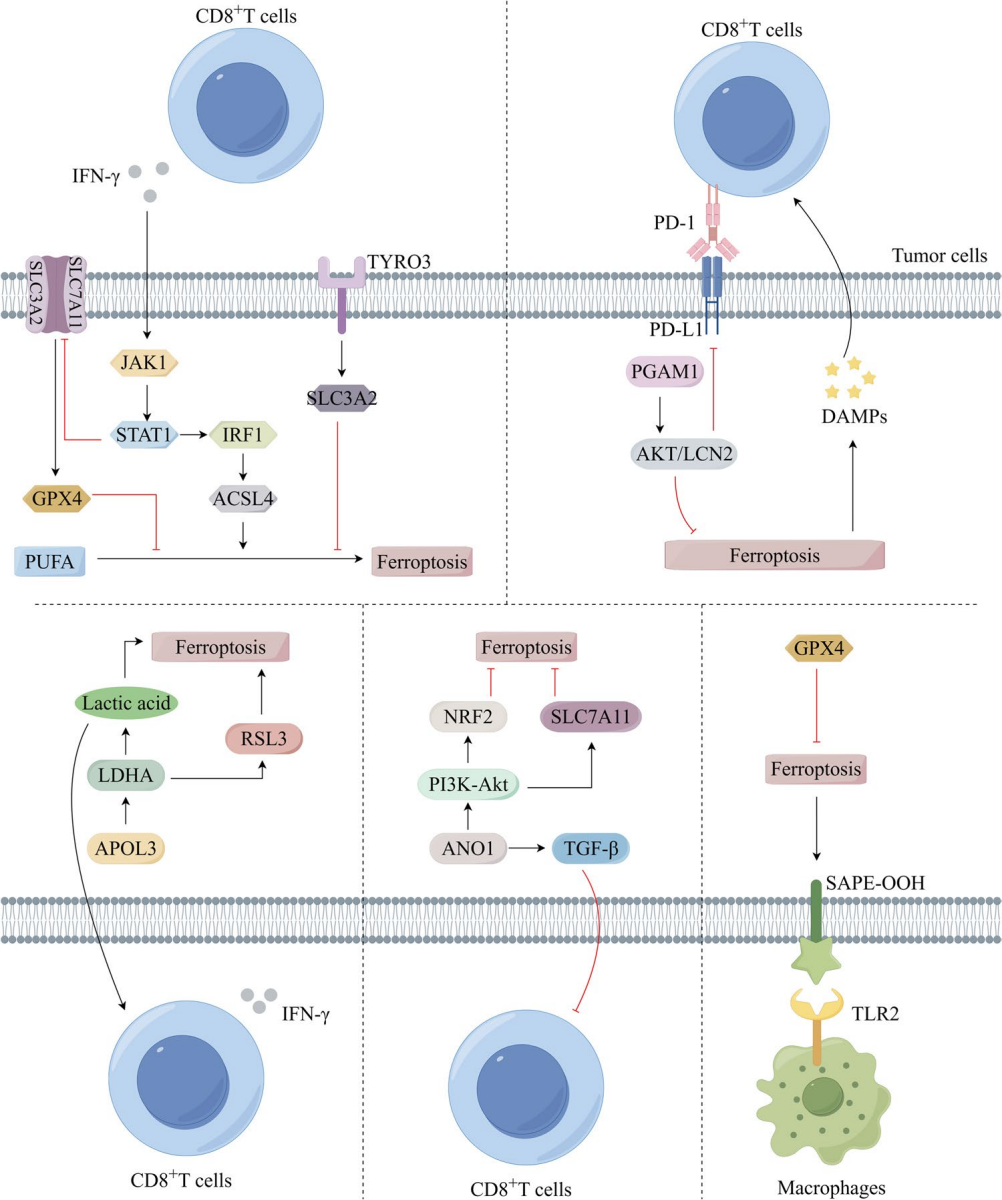
Enhancement of immunotherapy through ferroptosis in the tumour microenvironment. Ferroptosis is activated or inhibited in tumour cells by different pathways including the following: JAK1-STATA-IRF1 pathway, PGAM1-AKT-LCN2 pathway, APOL3-LDHA-RSL3 pathway, ANO1-PI3K-AKT pathway, and SAPE-OOH-TLR2 pathway, thereby promoting immunotherapy in the TME. This diagram was drawn by Figdraw (www.figdraw.com)

Immunotherapy is used in novel therapies to treat many types of cancer by harnessing the immune system to kill tumour cells [14]. The tumor microenvironment harbors a diverse array of immune cell types, including adaptive immune cells like CD8^+^T cells, CD4^+^T cells, regulatory T cells (Tregs), and B cells, as well as myeloid immune cells such as macrophages, dendritic cells (DCs), and myeloid-derived suppressor cells (MDSCs), and innate immune cells such as natural killer (NK) cells and innate lymphoid-like cells [15]. In the tumor microenvironment, immune cells vary substantially in composition and function even among patients with the same cancer type. These variations can be attributed to variables such as age, gender, body weight, and microbiota. There is evidence that tumor-associated immune cells contribute to tumor progression, although their effect can be contradictory. Tumor immunotherapy is a promising avenue for controlling tumor growth by regulating immune cells. While certain immune cells can inhibit tumor growth, others can promote it (Fig. 3). As a matter of fact, immunotherapy is highly dependent on immune cells functioning effectively within the microenvironment, especially in “hot” tumor types such as non-small cell lung cancer [16]. However, tumor cells in “hot” tumors typically exhibit an elevated expression of PD-L1 to facilitate their proliferation and metastasis. This overexpression allows them to bind to PD-1 on immune cells, thereby evading immune surveillance. On the other hand, “cold” tumors, such as pancreatic cancer, glioblastoma, and osteosarcoma, are characterized by a lower infiltration of immune cells. Consequently, these tumors exhibit a reduced expression of inhibitory immune checkpoint molecules since they do not require counteractive measures against immune cell surveillance and cytotoxicity [17]. The selection of immunotherapeutic approaches and the prognosis of tumor treatment heavily rely on the intricate and ever-changing nature of the tumor microenvironment (TME). Clinical tumor treatment has witnessed significant therapeutic prospects through tumor immunotherapy, encompassing adoptive cell therapy, immune checkpoint inhibitors, neoantigen vaccine therapy, and other modalities. The immunotherapy of tumor is described in detail in the reference [18].

## Crosstalk between ferroptosis and immune response in tumors

### Ferroptosis and immune cells

Ferroptosis has emerged as a promising approach in the field of tumor therapy, demonstrating significant antitumor efficacy in ferrotic tumor cells, albeit exhibiting a limited impact on certain malignant tumors. The tumor microenvironment encompasses a heterogeneous population of tumor cells and immune cells. Ferroptosis decreases the ability of immune cells, specifically T and B cells, to eliminate tumor cells, whereas inducing ferroptosis in tumor cells enhances the therapeutic potential of immunotherapy [19]. Immunotherapy and ferroptosis can therefore be combined to improve antitumor treatment. Depending on the immune microenvironment, tumors can be classified as “hot” or “cold”: hot tumors have a high number of immune T cells, while cold tumors have a low number of immune cells. In the case of “hot” tumors, the use of ferroptosis inducers as the primary therapeutic approach may result in a preferential elimination of immune cells rather than tumor cells. This selective effect can diminish the efficacy of inhibitory immune checkpoint blockers and potentially facilitate tumor progression [20]. Activating ferroptosis in “cold” tumors could enhance demise of tumors while targeting myeloid suppressor cells, Tregs, and M2-type macrophages selectively. Tumor immunotherapy can be enhanced through this process by ameliorating immunosuppressive conditions within the TME, which augments immune cell infiltration. Nanomaterials are increasingly being explored as potential mechanisms for controlling ferroptosis in tumor cells in recent years. It has been shown that this approach increases ferroptosis of tumor cells by promoting DC maturation. Combining this strategy with tumor immunotherapy has proven effective for triple-negative breast cancer, melanoma, and glioblastoma.

CD8^+^T cells, which serve as the primary cytotoxic cells against tumors, release IFN-γ to downregulate the expression levels of subunits SLC3A2 and SLC7A11 in the system Xc- [21]. Additionally, IFN-γ induces the binding of arachidonic acid (AA) to phospholipids on the membrane, resulting in the formation of AA-PLs by upregulating ACSL4 expression [22]. These two pathways collectively facilitate ferroptosis in tumor cells. High mobility group box-1 protein (HMGB1), phosphatidylethanolamine, and calreticulin are among damage associated molecular patterns (DAMPs) released by ferroptosis of tumor cells, which encourage DCs maturation and antigen presentation. By increasing the function of DCs, CD8^+^T cells are ultimately able to kill more tumors [23]. Moreover, DAMPs enhance immune responses against tumors by polarizing M1-type macrophages (TAMs) [24]. In contrast, ferroptosis induces PTGS2 expression, resulting in the production of PGE2 and the suppression of innate and acquired immune responses to tumors. Interestingly, cholesterol promotes the progression of CD8^+^T cells towards ferroptosis via fatty acid uptake via CD36, and the use of ferroptosis inducers may inhibit their activity as well [25]. Therefore, the relationship between ferroptosis and immunotherapy does not necessarily exhibit a positive regulatory effect. GPX4 depletion within the TME also causes ferroptosis, which inhibits CD8^+^T cell recognition and elimination of tumors. However, overexpression of GPX4 induces tumor cells to resist ferroptosis, thus diminishing its anti-tumor effects. Consequently, leveraging the inhibitory potential of ferroptosis as a supplementary approach to immunotherapy appears to be a viable strategy. In addition, Tregs may assist tumor cells in evading immune surveillance. Thus, both Treg reduction and ferroptosis promotion may enhance immunotherapy effectiveness. It is necessary to explore the relationship between Tregs and antitumor therapies in more detail [26]. GPX4 deficiency causes ferroptosis in M2 macrophages because nitric oxide synthase is not expressed. Conversely, macrophages of the M1 subtype exhibit high epitaxial expression of iNOS, leading to the release of NO with antioxidant properties, thereby reducing GPX4 dependency and susceptibility to ferroptosis. Through the STAT3 pathway, ferroptosis in tumor cells can promote fatty acid oxidation in macrophages, leading to their polarization towards the M2 subtype, which promotes tumor growth. Under high oxidative stress conditions within the tumor microenvironment, pancreatic ductal adenocarcinoma (PDAC) tumor cells secrete the KRAS G12D protein, which in turn stimulates fatty acid oxidation in macrophages and facilitates their polarization towards the M2-type through the STAT3 pathway [27]. The absence of the GPX4 and the use of ferroptosis inducers both lead to a reduction in M2-type macrophages and promote the repolarization of M2-type macrophages towards the M1-type [28]. AGE-mediated polarization of M2-type macrophages is observed in pancreatic cancer through excretion of KRAS-G12D by tumor cells during ferroptosis. Modulating the TME to attenuate the immune response is a crucial strategy. Moreover, DCs and NK cells are also prone to ferroptosis, which consequently confers resistance to immunotherapy in tumor cells. Activation of the SMAD pathway by macrophages through transforming growth factor β1 amplifies the vulnerability of tumor cells to ferroptosis. Additionally, ferrous tumor cells can impede the functioning of DCs, NK cells, and other immune cells by releasing lipid metabolites, thereby facilitating immune evasion. Interleukin 4 induced protein 1 (IL4I1) is an amino acid oxidase that plays a significant role in facilitating immune evasion of tumor cells through the enhancement of tryptophan metabolism [29]. Additionally, IL4I1 induces the production of indole-3-pyrubate (I3P), effectively scavenges free radicals in tumor cells, and inhibits oxidative stress and ferroptosis. Anti-PD-L1 antibody and exogenous PUFA intake have been found to effectively promote ferroptosis in tumor cells [30]. Furthermore, the interaction between ferrous tumor cells and immune cells within the TME is of great importance. Ferroptosis in tumor cells is induced through an inhibition of the transport of fatty acids from tumor cells to immune cells, as well as a promotion of immune cell regulation of ferroptosis.

Ferroptosis is hampered by the high intracellular expression of the system Xc- in MDSCs within the tumor microenvironment, coupled with the absence of the unsaturated fatty acid peroxidation pathway. Furthermore, the promotion of ferroptosis in polymorphonuclear-myeloid-derived suppressor cells (PMN-MDSCs) has been found to enhance T cell lipid uptake, induce ferroptosis in these cells, and suppress tumor immunity [31]. By inhibiting ferroptosis in PMNs, the transformation of immunosuppressive PMN-MDSCs can be avoided, leading to a reduction of immunosuppressive factors. Maintaining the integrity and functionality of the antioxidant pathway in anti-tumor immune cells can effectively enhance their ability to kill tumor cells. The compound NC06 has the ability to target MDSCs and induce ferroptosis through ASAH2, thereby improving the immunosuppressive environment [32]. Tumor infiltrating neutrophils (TINs) and PMN-MDSCs share similar functionalities and can facilitate tumor cell metastasis by the suppression of NK cells and effector T cells. TINs induce the up-regulation of siderophores via Acod1-dependent activation of sideroblasts and the Keap/Nrf2-associated antioxidant pathway [33]. Consequently, the inhibition or knockdown of Acod1 may synergistically enhance the effectiveness of immune checkpoint blockers by engaging a novel pathway, thereby bolstering immunotherapeutic efficacy in “hot” tumors.

### Ferroptosis-mediated immunotherapy

Ferroptosis exhibits a dual nature in the context of tumor immunotherapy, as it can induce impairments in the functionality of anti-tumor immune cells, including T cells and NK cells. Conversely, it can also trigger ferroptosis in tumor cells and tumor-promoting immune cells, such as M2-type macrophages, thereby augmenting the efficacy of immunotherapeutic interventions. Tumor cells can be targeted to undergo ferroptosis, which has potential benefits for immunotherapy (Fig. 3). The utilization of PD-L1 blockers elicits the secretion of IFN-γ by CD8^+^T cells, leading to the inhibition of transcription and expression of SLC7A11 and SLC3A2, thereby reducing GPX4 production via the JAK1/STAT1 pathway and facilitating ferroptosis in tumor cells [34]. Additionally, IFN-γ can stimulate ACSL4 expression through the STAT1/IRF1 pathway, catalyzing PUFA promoting ferroptosis [34]. However, CD8^+^T cells can also assume an antioxidant role by decreasing the M1/M2 macrophage ratio and enhancing SLC3A2 expression upon binding to TYRO3, which impedes tumor cell ferroptosis and fosters tumor progression. In various studies, PD-1 resistance has been observed [35]. These studies have demonstrated that cystatase not only inhibits GSH synthesis by degrading cystine and cysteine, thereby inducing ferroptosis, but also hinders the suppressive effects of immune checkpoints, thereby enhancing the anti-tumor immune response. Thus, immunotherapy and ferroptosis therapy are a promising combination following radiation therapy for tumors. Furthermore, the inclusion of ferroptosis inducers synergistically augments the efficacy of PD-L1 blockers, which target inhibitory immune checkpoints. The SAPE-OOH signaling pathway exhibited by ferrous tumor cells can also activate TLR2 receptors or compensatory mechanisms in macrophages, thereby enhancing the phagocytic activity of macrophages [36]. However, the GPX4 inhibitor, which acts as an antioxidant, displays greater sensitivity towards CD8^+^T cells and CD4^+^T cells, leading to a preferential induction of ferroptosis in killer T cells rather than tumor cells. Tissue immunotherapy tolerance may be developed by elevated PD-1 expression in tumor-infiltrating immune T cells compared with PD-L1 expression in tumor cells. The presence of PD-1 inhibitors in the tumor microenvironment promotes the activity of CD8^+^T cells. However, the intricate nature of tumors also results in a constrained immune response. DCs identify and exhibit tumor-associated antigens to CD8^+^T cells in reaction to signals related to lipid peroxidation associated with ferroptosis. This process stimulates the secretion of IFN-γ and enhances ferroptosis in tumor cells. Remarkably, mice subjected to a combined treatment of immunotherapy and ferroptosis inducers displayed robust anti-tumor effects. The induction of ferroptosis in tumor cells holds promise as an immunotherapeutic strategy. When magnetic nanoparticles are encapsulated with RSL-3, they can be used to precisely target tumor sites through the influence of external light beams. This targeting mechanism induces redox and metabolic disturbances within tumor cells, ultimately resulting in ferroptosis. PD-L1 blockers also facilitate T-lymphocyte infiltration into the tumor, which enhances immunotherapy effectiveness [37].

It is important to note that various tumor diseases necessitate distinct therapeutic regimens due to the presence of diverse disease-inducing factors. For instance, in cases of hepatocellular carcinoma, the reduction of GPX4 proves ineffective in inhibiting cancer cell proliferation and instead leads to the impairment of anti-tumor immune cell functionality. The phenomenon can be attributed to the immunosuppressive TME shaped by MDSCs. Ferroptosis inducers and HMGB1 blockers can effectively suppress the growth and metastasis of hepatocellular carcinoma cells. By targeting the inhibition of PGAM1, the AKT/LCN2 pathway is inhibited, leading to the promotion of ferroptosis through the accumulation of intracellular ROS. Additionally, PGAM1 inhibits PD-L1, enhancing the immune response against tumors [38]. Furthermore, the inhibition of APOC1 expression promotes ferroptosis in M2-type macrophages and induces their repolarization towards M1 macrophages. Within the tumor microenvironment, APOC1 expression is positively correlated with CD8^+^T cells and NK cells that are anti-tumor immune cells. Through the inhibition of immune checkpoint blockers, APOC1 further modifies the immune microenvironment, improving the effectiveness of anti-tumor immunotherapy. Poor prognosis is associated with low APOL3 expression in colorectal cancer. APOL3 plays a role in the ubiquitination of LDHA, thereby increasing tumor cell sensitivity to the ferroptosis-inducing protein RSL3. Additionally, the APOL3-LDHA pathway facilitates the expression of IFN-γ, thereby augmenting anti-tumor immunity [39]. The promotion of FBXO10 protein transcription and expression, the induction of ACSL4 degradation, the inhibition of ferroptosis are facilitated by the PKC pathway through the action of CYP1B1-derived 20-HETE. The combination of CYP1B1 inhibition and inhibitory immune checkpoint blockers effectively enhances anti-tumor immunotherapy. Upon the failure of immunotherapy, the ANO1 gene is upregulated, activating the PI3K-Akt pathway, resulting in the expression of NRF2/SLC7A11 proteins and inhibiting oxidative stress in tumor cells. Furthermore, ANO1 has been shown to stimulate the expression of TGF-β, facilitate the aggregation of tumor-associated fibroblasts, suppress the immunoreactivity of CD8^+^T cells, and decrease the secretion of TNF-α, granzyme B, and IFN-γ. Proliferation and metastasis of tumors are ultimately accelerated by these effects. In the context of bladder cancer, the mitochondria-targeting drug BQR liposome (BQR@MLipo) specifically targets the mitochondria of tumor cells. This targeted approach leads to the release of buchinha, which inhibits the activity of the mitochondria-specific antioxidant DHODH. Consequently, lipid peroxidation and ferroptosis are induced. This information was reported in a study [40]. Moreover, the liberation of mitochondrial DNA (MtDNA) alongside DAMPs like ATP triggers the initiation of the cGAS-STING pathway and associates with the receptors found on diverse antigen-presenting cells. This process amplifies the recognition and presentation of antigens by DCs and macrophages. In the context of brain cell tumors, the coadministration of ferroptosis inhibitors and inhibitory immune checkpoint blockers diminishes the infiltration of TAMs and fosters the repolarization of macrophages towards the M1 phenotype. Consequently, this intervention augments the infiltration of tumors and enhances the cytotoxic potential of CD8^+^T cells [41]. KEAP1 is specifically methylated by protein arginine methyltransferase 5 (PRMT5) in triple-negative breast cancer cells, inhibiting intracellular iron transport. In the context of immunotherapy for TNBC, the combination of PRMT5-targeted inhibitors could effectively enhance the induction of ferroptosis in TNBC by CD8^+^T cells, thereby promoting cell death [42]. Similarly, in prostate cancer cells, the heterogeneous nuclear ribonucleoprotein L (HnRNP L) induces the stable expression of YY1, which in turn upregulates the production of PD-L1. There is an inverse relationship between upregulation of PD-L1 expression and PD-L1 expression itself. Inhibition of HnRNP L promotes anti-tumor immune function and CD8^+^T cell-induced ferroptosis of tumor cells.

### Combination of ferroptosis inducers and immunotherapeutic agents

#### Ferroptosis inducers (concomitant immunotherapy)

Tumor cell ferroptosis can be induced by ferroptosis inducers through various pathways, and the activation of ferroptosis-based TME immune responses can occur through multiple pathways as well. Additionally, the immune response can further augment tumor cell ferroptosis through positive feedback, thereby reinforcing the efficacy of ferroptosis inducers. Ferroptosis inducers have demonstrated efficacy in producing anti-tumor effects in various types of cancers, including TNBC, melanoma, glioblastoma, as evidenced by the findings presented in Table 1. Specifically, in the case of bladder cancer, the use of BQR@MLipo has been shown to effectively target and inhibit DHODH activity, leading to mitochondrial lipid peroxidation and subsequent induction of ferroptosis in bladder cancer cells. Through the cGAS-STING pathway, DAMPs, such as calreticulin, ATP, and HMGB1, promote DCs and macrophages’ recognition of tumor antigens and facilitate the infiltration of CD8^+^T cells into tumors, thereby stimulating tumor immunity [40]. The administration of COF-919 induces ferroptosis in TNBC by promoting the accumulation of ROS and lipid peroxidation, while simultaneously reducing GSH and GPX4 expression. TME immunosuppression is improved with DAMPs since they induce DC maturation and CD8^+^T cell infiltration into tumors, suppress Treg and MDSC activity, and suppress M2-type macrophage function [43]. Additionally, PAMAM not only affects the function of GPX4, but also inhibits the expression of FSP1, thereby inducing ferroptosis [44]. MTX-LDH@MnO2 induces ferroptosis by inhibiting GPX4 and BH4 expression [45]. Similarly, FeGd-HN@TA-Fe^2+^-SN38 nanoparticles have been shown to induce the Fenton reaction and endoplasmic reticulum stress in tumor cells, leading to lipid peroxidation and ferroptosis. Additionally, the release of exosomes containing damaged DNA has been found to induce DCs maturation through the cGAMP-STING-TBK1 pathway, while the secretion of IFN-β activates CD8^+^T cells, resulting in the release of IFN-γ and subsequent inhibition of GSH and GPX4 expression in tumor cells [46]. Furthermore, the compound TCPP-TK-PEGPAMAM-FA has been shown to stimulate lipid peroxidation and ferroptosis in tumor cells by inhibiting the HIF-1α pathway and subsequent endogenous lipid droplet biosynthesis [47]. In osteosarcoma, however, Cu/ZIF-8@U-104@siNFS1-HA induces ferroptosis in tumor cells, resulting in the polarization of macrophages to the M1 type and enhanced immune response against tumors [48]. In the case of melanoma, the compound TPL@TFBF, which does not rely on GPX4, induces tumor cell ferroptosis through the NRF2 pathway, thereby promoting immune responses and inhibiting tumor proliferation and metastasis [49]. The metal-phenolic networks nanoplatform exhibits dual functionality by inducing ferroptosis in tumor cells and inhibiting PD-L1 expression, thereby enhancing anti-tumor immune responses [50]. Nevertheless, ferroptosis inducers may negatively affect normal cells as well as antitumor immune cells within the TME, resulting in reduced effectiveness. To elucidate how these drugs target tumor cells in the TME, further investigation is needed. Additionally, future studies should explore the possibility of enhancing the precision targeting of ferroptosis inducers to tumor cells through the use of dual material coating. The initial substance facilitates the discharge of the ferroptosis inducer within the TME, whereas the subsequent substance facilitates the discharge of the ferroptosis inducer within the tumor cells via the tumor antigen, thereby mitigating the deleterious impact of the ferroptosis inducer on non-tumor cells.

**Table 1:** Antitumor effects of ferroptosis inducers (concomitant immunotherapy)

Authors	Drugs	Tumor types	Therapeutic mechanisms	References
Ding Q, et al.	BQR@MLipo	Bladder cancer	Inhibits of DHODH activity, induces lipid peroxidation and ferroptosis in tumor cells. Release of DAMPs induces the cGAS-STING pathway to enhance DC cells maturation.	[40]
Zhang L, et al.	COF-919	TNBC	Promotes the production of ROS, induces lipid peroxidation and ferroptosis. Enhances the infiltration capacity of CD8^+^T cells to remodel TME.	[43]
Guo S, et al.	FeGd-HN@TA-Fe^2+^-SN38 nanoparticles	TNBC	Induces ferroptosis in tumor cells. The release of IFN-β and IFN-γ stimulates the activation of NK cells and CD8^+^T cells.	[46]
Wang WJ, et al.	FerroceneAppended Iridium(III) Diphosphine Complex	TNBC	Induces of lipid peroxidation and ferroptosis in tumor cells, promotes the release of DAMPs and enhances immunogenic cell death in tumor.	[51]
Wang Z, et al.	Cu/ZIF-8@U-104@siNFS1-HA	Osteosarcoma	Inhibits GSH and GPX4 expression, promotes tumor ferroptosis. Increases M1-type macrophage polarisation, induces antigen-presenting cells maturation, inhibits Treg cells function and promotes tumor infiltration of CD8^+^T cells.	[48]
Han W, et al.	ZnP@DHA/Pyro-Fe core-shell nanoparticles	Colon cancer	Induces ROS generation and ferroptosis in tumor cells. Release of DAMPs induce DC cells maturation and enhance tumor infiltration of CD8^+^T cells.	[52]
Li LG, et al.	DHA@MIL-101	Lung cancer	Induces ROS accumulation, lipid peroxidation and ferroptosis in tumor cells. Release of DAMPs activate NF-κB through the cGAS/STING pathway and induces M1-type repolarisation of TAM, which also stimulate DC cells maturation.	[53]
Han N, et al.	Dihydroartemisinin	Lung cancer	Inhibits of GPX4 expression, induces lipid peroxidation and ferroptosis. Subsequent endoplasmic reticulum stress and damaged DNA fragments improve anti-tumor immune responses.	[54]
Liang JL, et al.	HBMn-FA	TNBC	Induces of oxidative stress and ferroptosis in tumor cells, and release of DAMPs activate the cGAS-STING pathway to further promote DC cells maturation.	[55]
Zhang Q, et al.	TCPP-TK-PEGPAMAM-FA	TNBC	Inhibits the HIF-1α pathway, enhances lipid peroxidation and ferroptosis. Released DAMPs and antigens stimulate anti-tumor immune responses.	[47]
Zhou Y, et al.	PAMAM	TNBC	Blocks GPX4 and FSP1 expression and induces lipid peroxidation and ferroptosis. Released DAMPs enhances DC cells maturation and infiltration of CD8^+^T cells into the TME.	[44]
Liu Z, et al.	MTX-LDH@MnO_2_ nanoplatform	TNBC	Inhibits BH4 biosynthesis, depletes GSH and GPX4 and promotes ferroptosis. Release of DAMPs further induce anti- tumor immune responses.	[45]
Xie B, et al.	FeAMV	TNBC	Inhibits the function of the xCT system, GSH and GPX4, induces ROS accumulation and promotes ferroptosis. Antigens and DAMPs released after ferroptosis can promote anti- tumor immune responses.	[56]
Song WF, et al.	Self-assembled copper-alanine nanoparticles	TNBC	Induces the Fenton reaction and depletes GSH, promotes a massive build-up of ROS and induces ferroptosis. ROS also activates the anti- tumor immune response.	[57]
Yu Y, et al.	MFE-NCPs	Colon cancer	Inhibits of BH4 biosynthesis, promotes oxidative stress and ferroptosis. DAMPs released by ferroptosis in tumor cells enhance anti- tumor immune responses.	[58]
Li K, et al.	Cu_2 − x_Se/ZIF-8@Era-PEG-FA	TNBC	Inhibits GPX4 and GSH expression, induces ferroptosis. Reduction of miR301 in tumor cell exosomes promotes M1-type repolarisation of TAM. Activation of CD8^+^T cells and secretion of IFN-γ induce ferroptosis in tumor cells.	[59]
Wang S, et al.	TPL@TFBF	Melanoma	Promotes intracellular ROS production in tumor cells and induces lipid peroxidation and ferroptosis through Fenton reaction and inhibition of NRF2-related pathways. Released DAMPs induce anti-tumor immune responses.	[49]
Liu P, et al.	Metal-phenolic networks nanoplatform	Melanoma	Increases lipid peroxidation and ferroptosis in tumor cells, released DAMPs promote anti- tumor immune responses. T-cell-derived IFN-γ hinders the function of the xCT system in tumor cells. MPNs also blocks PD-L1 protein expression in tumor cells.	[50]
Xie L, et al.	PFG-MPNs	Melanoma	Induces Fenton reaction, lipid peroxidation and ferroptosis in tumor cells, release of DAMPs promote DC cells activation. T-cell-derived IFN-γ hinders xCT system function as well as GSH and GPX4 expression in tumor cells.	[60]
Lei L, et al.	ZN-FU MNS	Colon cancer	Damages mitochondria and produces ROS, induces lipid peroxidation and ferroptosis. Release of DAMPs activate DC cells and promote anti-tumor immune responses.	[61]
Pei Z, et al.	VS_2_-PEG	Colon cancer	Depletes intracellular GSH and GPX4, induces ferroptosis of tumor cells, promotes IL-1β efflux, inhibits pro-tumor immune cells function, promotes repolarisation M1-type macrophages.	[62]
Ruan Y, et al.	E. coli@Cu_2_O microbial nanohybrid	Colon cancer	Reduces GSH and GPX4 activity, induces lipid peroxidation and ferroptosis. Release of DAMPs promote anti-tumor immune response.	[63]
Deng X, et al.	Ca & Mn dualion hybrid nanostimulator	TNBC	Reduces GSH and GPX4 activity, induces lipid peroxidation and ferroptosis. Release of antigen activates STING signalling activation, promotes M1-type polarisation of TAM and DC cells maturation.	[64]
Wang H, et al.	PEGylated Manganese–Zinc Ferrite Nanocrystals	Prostate cancer	Inhibits GSH and GPX4 activity and promotes ferroptosis. Release of IFN-γ and antigen synergistically promote anti-tumor immune responses.	[65]
Li Q, et al.	Fe_3_O_4_-DHJS@HRM nanoparticles	Osteosarcoma	Promotes Fenton reaction, inhibits NRF2 pathway-related antioxidant functions, and induces lipid peroxidation and ferroptosis. Release of DAMPs induces anti-tumor immune responses, induces M1-type polarisation of TAM.	[66]
Chen M, et al.	D@FMN-M	TNBC	Depletes GSH and GPX4 expression, triggers Fenton reaction and releases ROS, induces ferroptosis in tumor cells. Release of tumor cell-associated antigens induce activation of anti-tumor immune responses.	[67]
Liu B, et al.	Fe_3_O_4_-siPD-L1@M_− BV2_	Glioblastoma	Induces ferroptosis and reduces PD-L1 expression in tumor cells and releases tumor-associated antigens that induce anti-tumor immune responses.	[68]
Yang N, et al.	CFA/PRV@MM	TNBC	Depletes GSH and GPX4 expression, inhibits the antioxidant capacity of FSP1/CoQ10 and induces lipid peroxidation and ferroptosis in tumor cells. Released DAMPs induce DC cells maturation and M1-type macrophage polarisation.	[69]
Yang Q, et al.	CP nanoformulation	Melanoma	Promotes ferroptosis, blocks PD-L1 expression by inhibiting the NF-κB pathway and p38/MAPK pathway, and enhances anti-tumor immune responses.	[70]
Liu J, et al.	IFNγ/uMn-LDHs	TNBC	Depletes GSH and GPX4 expression, inhibits the function of the xCT system and promotes ferroptosis in tumor cells. Release of DAMPs induce an anti-tumor immune response.	[71]

#### Immunotherapeutic drugs (accompanied by ferroptosis of tumor cells)

Immunotherapeutic medications have the potential to enhance tumor cell ferroptosis by stimulating immune cell activation and releasing substances, such as IFN-γ, to impede the antioxidant function of tumor cells. As a result of using S-biAb/dEGCG@NPs in glioblastoma, CD8^+^T-cells were infiltrated into the TME. This led to the release of IFN-γ, which effectively inhibited the expression of SLC3A2 and SLC7A11 in tumor cells. Consequently, there was a reduction in the levels of GSH and GPX4, ultimately promoting ferroptosis [72]. Conversely, in hepatocellular carcinoma, the application of CH-OD-SSZ hydrogel triggered the release of chemokines, including NLRP3, IL-1β, and TNF-α, via the MAPK pathway. During this process, M1-type macrophages polarized and DCs within the TAM population matured. Collectively, these events facilitated immune responses within tumor cells and had the potential to induce immunogenic ferroptosis [73]. There is evidence that immunotherapeutic medications facilitate the ability of immune cells to infiltrate tumors, and the combination of inhibitory immune checkpoint inhibitors also enhances the antitumor effects. Immunotherapeutic drugs solely necessitate targeting the TME rather than anti-tumor immune cells, thereby diminishing the intricacy and expense of drug manufacturing while concurrently heightening drug safety.

#### Combination of immunotherapeutic agents and ferroptosis inducers

The concurrent utilization of immunotherapeutic drugs and ferroptosis inducers demonstrates a synergistic impact on suppressing tumor growth. This effect is achieved through the induction of ferroptosis via diverse pathways and the activation of immune responses through distinct mechanisms. This combined approach has exhibited efficacy in various types of cancer, including melanoma, hepatocellular carcinoma, and TNBC (Table 2). In the case of colon cancer, the application of Fish oil-based microemulsion has been observed to obstruct the PD-1/PD-L1 pathway, augment the infiltration of CD8^+^T cells within the TME, and stimulate the release of IFN-γ, thereby promoting the induction of ferroptosis in tumor cells. On the contrary, the administration of fish oil results in an excessive accumulation of PUFA in tumor cells, thereby facilitating ferroptosis. The immune response triggers ferroptosis in tumor cells, while tumor cell ferroptosis reciprocally enhances the immune response, thereby synergistically augmenting the antitumor efficacy [74]. In the context of TNBC, the utilization of MOF@GOx@MnO2@PEG: MGMP could induce tumor cell ferroptosis by suppressing the expression of tumor cell GSH and GPX4. Additionally, PD-L1 antibodies can strengthen the immune response and exert anti-tumor effects [75]. A hybrid nanoparticle, siProminin2@PSN-FeNP, increases the immune response against tumors by working with oxaliplatin to reduce iron efflux from tumor cells, thus enhancing ferroptosis. Melanoma cells were shown to undergo ferroptosis through the Keap1-NRF2-heme oxygenase 1 (Hmox1) pathway when Gel@WA-cRGD inhibited GSH and GPX4 expression. Additionally, the combination of PD-L1 has been observed to augment the antitumor effect [76]. Moreover, fe@OVA-IR820 has been found to synergistically exert an antitumor effect when combined with CTLA-4 antibody [77]. The variation in tumor types and TME necessitates the identification of rational and stable therapeutic formulations for different types of tumors when utilizing a combination of ferroptosis inducers and immunotherapeutic agents. Additionally, it is crucial to exercise caution in order to prevent the impairment of antitumor immune cells by ferroptosis-inducing agents, as this may hinder the efficacy of immunotherapeutic drugs.

**Table 2:** Antitumor effects of immunotherapeutic drugs in combination with ferroptosis inducers

Authors	Drugs	Tumor types	Therapeutic mechanisms	References
Yang X, et al.	Fish oil-based microemulsion	Colon cancer	Enhances TME infiltration capacity of CD8^+^T cells, release of IFN-γ exerts anti-tumor effects, and fish oil directly induces ferroptosis in tumor cells.	[74]
Zhang K, et al.	MOF@GOx@MnO_2_ @PEG: MGMP	TNBC	Accelerates GSH depletion, increases H_2_O_2_ content and promotes ferroptosis of tumor cells. Synergistic inhibitory immune checkpoint blocker enhances anti-tumor ability.	[75]
Shi W, et al.	Alum-CpG@Fe-Shikonin NPs	TNBC	Activates ferroptosis of tumor cells, releases DAMPs and antigens to accelerate the maturation of anti-tumor immune cells, induces TAM repolarisation to M1-type and enhances anti-tumor immunity.	[78]
Ling YY, et al.	Ferrocenecontaining Ir(III) photosensitizer (IrFc1)	TNBC	Targets ferrous tumor cells and promots oxidative stress-induced ferroptosis via transferrin receptor, activates the immune response of CD8^+^T cells.	[79]
Cheng Z, et al.	Gel@WA-cRGD	Melanoma	Inhibits GPX4 and GSH expression and induces ferroptosis in tumor cells via the Keap1-NRF2-Hmox1 pathway. Release of DAMPs and antigen induce antigen-presenting cells maturation. Anti-PD-L1 antibody enhances anti-tumor immune response.	[76]
Wang Y, et al.	hybrid nanoparticle siProminin2@PSN-FeNP	TNBC	Blocks exosome release, reduces tumor cell iron efflux and increases ferroptosis. Synergises with oxaliplatin to promote anti-tumor immune responses.	[80]
Dai X, et al.	RSL-3 + PD-1@gel	Liver cancer	Inhibits GPX4 expression, promotes oxidative stress and ferroptosis in tumor cells. Release of antigen promote anti-tumor immune responses. Release of PD-1 reduces recognition barriers in CD8^+^T cells.	[81]
Chin YC, et al.	Fe_3_O_4_@Chl/Fe CNPs	Bladder cancer	Reduces GSH and GPX4 levels, and induces lipid peroxidation and ferroptosis. Inhibits protein function of PD-L1 and reduces of M2-type macrophages.	[82]
Guo W, et al.	AuNp-miR-21–3p	Melanoma	Promotes lipid peroxidation and ferroptosis in tumor cells. Inhibitory immune checkpoint blockers coordinate anti-tumor effects.	[83]
Ma S, et al.	Fe@OVA-IR820	Melanoma	Down-regulates GPX4 expression and promotes ferroptosis in tumor cells. Released DAMPs induce anti-tumor immune responses and synergise with anti-CTLA-4 antibodies to exert anti-tumor effects.	[77]
Hou G, et al.	Hydrazide/Cu/Fe/ indocyanine green coordinated nanoplatform	Melanoma	Promotes Fenton reaction, inhibits GSH and GPX4 activity, and induces lipid peroxidation and ferroptosis in tumor cells. Released DAMPs promote anti-tumor immune response in concert with anti-PD-1 antibody.	[84]
Lei H, et al.	MnMoOx NPs	Colon cancer	Reduces GSH and GPX4 activity in tumor cells, induces lipid peroxidation and ferroptosis. Released DAMPs induce anti-tumor immune responses.	[85]
Bao Y, et al.	FG-CDs@Cu	Colon cancer	Depletes intracellular GSH and GPX4 in tumor cells, generates ROS, induces lipid peroxidation and ferroptosis. Reduction of HIF-1α effectively stimulates the conversion of M2-type macrophages to M1-type, and release of DAMPs enhance the anti-tumor immune response.	[86]
Li Q, et al.	Leukocyte membrane coated poly encapsulating glycyrrhetinic acid	Colon cancer	Promotes Fenton reaction in tumor cells and inhibits GSH and GPX4, induces lipid peroxidation and ferroptosis. Combination of glycerol ferulate, GCMNPs and anti-PD-L1 antibodies synergistically enhance anti-tumor immune responses.	[87]

## Summary and prospects

Over the past three years, researches have predominantly focused on the utilization of combined anti-tumor therapy involving ferroptosis therapy and immunotherapy. The main goal of this approach is to induce ferroptosis in tumor cells, resulting in the release of DAMPs from the cells. DAMPs stimulate DCs maturation and promote CD8^+^T cell and NK cell antitumor responses through antigen presentation. Further, CD8^+^T cells can be enhanced to recognize tumor cells with the implementation of inhibitory immune checkpoint blockers. This comprehensive strategy has exhibited significant efficacy in suppressing tumor development across a diverse range of oncology experiments. Ferroptosis induction in anti-tumor immune cells remains to be determined when high concentrations of ferroptosis inducers are introduced. This phenomenon could potentially result in diminished therapeutic effectiveness, necessitating higher drug dosages and potentially causing adverse effects. Therefore, further advancements are required to enhance the precision of ferroptosis inducers in targeting tumor cells. Future research should focus on the development of tumor cell therapy strategies that specifically enhance the functionality of anti-tumor immune cells within the TME, while also coordinating the stimulation of tumor-promoting cells and inducing ferroptosis in tumor cells. By inhibiting diverse negative feedback mechanisms within the TME, immune cells cannot initiate ferroptosis via cytokines and other signaling pathways after tumor cells have ferroptosed. Tumor-specific antigens can serve as binding sites for ferroptosis inducers, enabling precise targeting of tumor cells, with the release of ferroptosis inducers occurring exclusively upon phagocytosis by tumor cells. This approach holds promise for enhancing therapeutic targeting efficacy while minimizing undesirable side effects. Furthermore, the induction of ferroptosis has demonstrated efficacy in enhancing the immunosuppressive state within the TME. Consequently, the integration of chimeric antigen receptor T-cells (CAR-T) or T cell receptor-gene engineered T cells (TCR-T) therapies with ferroptosis inducers presents a promising therapeutic approach. Moreover, the implementation of gene editing technology can mitigate T-cell sensitivity to ferroptosis and minimize the interference of ferroptosis inducers with the immune response. Notably, the mechanism by which NK cells act upon tumors differs from that of T cells, thus highlighting the potential value of exploring CAR-NK cell therapies in diverse TMEs. The efficacy of tumor therapy relies on the enduring presence of immune cells and their consistent ability to eliminate tumor cells. TME can vary significantly among individuals, necessitating the development of distinct therapeutic protocols tailored to specific TME. These protocols aim to enhance immune responses and establish highly effective and enduring therapeutic strategies, which constitute the fundamental principles of antitumor therapy.

With the rapid advancement of analytical technologies, the utilization of high-resolution techniques such as spatial histology has enabled a deeper understanding of the intricate interactions occurring within TME. This includes elucidating the coordination between tumor cell ferroptosis and immune cell therapy, which collectively contribute to the exertion of antitumor effects. Cancer-associated fibroblasts (CAFs) represent the most prevalent component of the TME, which is made up of tumor cells, immune cells, and fibroblasts. Due to metabolic demands, both tumor cells and CAFs release lactate, resulting in a weakly acidic environment (pH 5.5–6.6) within the TME. Lactate, a significant carbon source utilized for lipid synthesis, plays a crucial role in the promotion of MUFA production and the inhibition of ACSL4 through the hydroxycarboxylic acid receptor 1 (HCAR1)/ monocarboxylate transporter 1 (MCT1)- sterol regulatory element binding protein 1 (SREBP1)- stearyl coA desaturase 1 (SCD1) pathway, thereby inhibiting the ferroptosis in tumor cells. The CAF also induces ferroptosis in NK cells and facilitates tumor cells’ resistance to immune response. Firstly, the up-regulation of iron regulatory genes, such as ferritin 1 and heparin, in CAFs enhances the efflux of iron ions to NK cells. Secondly, CAFs stimulate the expression of NCOA4 in NK cells via follicle suppressor-like protein 1 (FSTL1), leading to ferritin autophagy and subsequent ferroptosis in NK cells. CAFs have been found to exert their protective effects on tumor cells by targeting arachidonate-15-lipoxygenase (ALOX15) and releasing cysteine and GSH to counteract oxidative stress and ferroptosis.

Autophagy plays a crucial role in cell survival and the regulation of iron homeostasis. In the context of pancreatic cancer, autophagy facilitates the secretion of IL-6 from tumor cells, up-regulates FPN expression in CAF, leading to enhanced iron efflux, and subsequently increases the synthesis of LIP and ISC in tumor cells. This process also promotes the expression of succinate dehydrogenase complex iron sulfur subunit B (SDHB), thereby improving mitochondrial function and supporting tumor development. Conversely, in conditions of autophagy deficiency, reduced levels of LIP result in decreased SDHB expression and compromised mitochondrial function. Iron supplementation or ectopic expression of SDHB or ISC assembly 1 (ISCA1) can improve mitochondrial defects and promote tumour progression [88]. Furthermore, the degradation of FPN autophagy through the selective autophagy receptor nuclear receptor coactivator 4 (NCOA4) leads to the release of iron, which helps maintain the LIP in the cytosol and supports the synthesis of ISC proteins to maintain mitochondrial homeostasis. In pancreatic cancer, the upregulation of ferritin autophagy mediated by NCOA4 enhances iron metabolism and promotes tumour growth [89]. However, elevated Fe^2+^ may also promote ferroptosis in tumour cells to exert anti-tumour effects. Suppression of autophagy-related genes ATG5 and ATG7 has been shown to decrease intracellular Fe^2+^ levels and attenuate ferroptosis. The interplay between autophagy and ferroptosis represents a promising avenue for future investigation. Cuproptosis, a recently proposed phenomenon in 2022, is distinguished by the copper-dependent accumulation of lipoylated proteins and represents a novel regulated mechanism of cellular demise. Intracellularly, GSH effectively chelates copper ions, thereby mitigating cuproptosis, while concurrently inhibiting lipid peroxidation to attenuate ferroptosis. GSH within cells serves as a pivotal focal point for intercommunication between ferroptosis and cuproptosis, as the depletion of GSH induced by ferroptosis or the GSH complexation reaction during cuproptosis fosters an amplifying effect on the alternate cell death process. As an example, inhibiting GSH synthesis and ferredoxin 1 degradation facilitates the accumulation of lipoylated proteins and cuproptosis by inducing ferroptosis. The concurrent utilization of both mechanisms of cell death could be contemplated as a means to augment the eradication of tumor cells. Alternatively, it is worth investigating whether tumor cells that are unresponsive to ferroptosis can be targeted using cuproptosis, and vice versa.

It is equally important to understand the significance of the “stimulus responsiveness” of the TME along with the synergistic potential of ferroptosis and immunotherapy. Addressing the specific targeting of the TME and mitigating systemic toxicities are imperative considerations in antitumor therapy. Conventional chemotherapy lacks precise tumor targeting, leading to indiscriminate cell death in both malignant and healthy cells. Conversely, the utilization of specialized nanomaterials exhibits a targeted effect on the TME, thereby circumventing adverse effects on normal cells and minimizing the impact on patients’ prognoses. The acidic microenvironment (approximately pH 5.5) resulting from the heightened metabolic activity of tumors facilitates the degradation or cleavage of nanomaterials sensitive to pH. This process enables the release of tumor therapeutic drugs such as Fe^2+^, cisplatin, adriamycin, and artemisinin, among others, thereby enhancing the efficacy of tumor therapy. It is important to highlight that the acidity in normal cellular lysosomes (pH approximately 4.5) surpasses that of the TME. Consequently, the pH sensitivity of nanomaterials must be carefully regulated during their design. The requirements for drug release from nanomaterials must adhere to two specific criteria: firstly, they must align with the acidic range commonly found in TME, while simultaneously excluding the acidic range present in normal cellular and lysosomal environments. Additionally, the elevated levels of ROS in tumor cells can be targeted by nanomaterials. The ROS-induced cleavage of chemical double bonds facilitates the release of antitumor drugs, such as GSH scavengers, diethyl maleate (DEM), and monocarboxylic acid transporter 4, which in turn inhibit siRNAs. In the context of increased levels of ROS, GSH is also elevated to preserve the equilibrium within the TME. Various materials, including cancer cell membrane-coated nanocarriers and pyrite nanoenzymes constructed from metal-organic frameworks and glucose oxidase (GOX), have the ability to release antitumor drugs such as cinnamaldehyde and irons in response to elevated GSH levels. Insufficient blood supply and disrupted vasculature resulting in a high metabolic rate contribute to the development of hypoxic conditions within the TME. Nanocarriers incorporating azobenzene linkers have the capability to liberate antitumor drugs, including GSH scavengers, sorafenib, and ferritin, in hypoxic environments. It is important to acknowledge that the effective delivery of these nanomaterials to tumors necessitates circumventing specific circumstances, such as acidic conditions in the skin, heightened ROS levels resulting from other diseases, increased GSH due to liver proliferation, and cellular hypoxia in diverse conditions. Furthermore, it is imperative to acquire a more extensive and intricate comprehension of the stage-specific metabolic mechanisms exhibited by distinct tumors. This knowledge is crucial in order to ascertain the optimal selection of nanomaterials and ensure that the drugs encapsulated within them can effectively target the tumor microenvironment, ultimately leading to the efficient eradication of malignant cells.

## Data Availability

No datasets were generated or analysed during the current study.
